# Gastrointestinal Stromal Tumor (GIST) Causing Obscure Gastrointestinal Bleeding: An Uncommon Way of Diagnosing An Uncommon Disease

**DOI:** 10.7759/cureus.9558

**Published:** 2020-08-04

**Authors:** Karolina N Dziadkowiec, Peter Stawinski, Sergio A Sánchez-Luna, Aviv Katz

**Affiliations:** 1 Internal Medicine, University of Miami, John F. Kennedy Regional Campus, Atlantis, USA; 2 Center for Advanced Therapeutic Endoscopy / Division of Gastroenterology, Hepatology & Nutrition, Allegheny Health Network / Allegheny Center for Digestive Health, Pittsburgh, USA; 3 Gastroenterology and Hepatology, John F. Kennedy Medical Center, Atlantis, USA

**Keywords:** gastrointestinal stromal tumor (gist), obscure gastrointestinal bleeding, endoscopy

## Abstract

Gastrointestinal stromal tumors (GISTs) are neoplasms that arise from the wall of the gastrointestinal tract or, rarely, from other intra-abdominal tissues. They are the most common mesenchymal tumors of the gastrointestinal tract and they should be considered in the differential diagnosis of obscure gastrointestinal bleeding. Computed tomography angiogram (CTA) can be utilized as an alternative imaging study when endoscopic and colonoscopy results are non-diagnostic. We report a case of a 59-year-old woman who presented with recurrent episodes of obscure overt gastrointestinal bleeding secondary to a gastrointestinal stromal tumor (GIST).

## Introduction

Gastrointestinal stromal tumors (GISTs) originate from gastrointestinal mesenchymal tissue, which predominantly consists of spindle cells, epithelioid cells, and polymorphic cells. Interstitial cells of Cajal (ICC) are recognized as the precursor cells of GISTs. ICCs are considered the pacemaker cells of the gastrointestinal tract and are immunostained by antibodies against CD117. Similarly, CD117 and CD34 are important histopathological biomarkers of GISTs. The most common presenting symptoms of GISTs are gastrointestinal bleeding, abdominal pain, distension, and discomfort due to possible tumor-induced mass effect. GISTs have no specific endoscopic findings, and diagnosis involves immunohistochemical analysis of KIT, CD34, or DOG1, essential for a definitive diagnosis [1]. Surgical resection is the first line of therapy for resectable GISTs without metastasis; whereas the primary approach for unresectable, metastatic, or recurrent GISTs is the use of tyrosine kinase inhibitors [2]. A GIST is considered to be a potentially malignant tumor, and these are not classified as either benign or malignant but are rather stratified using the widely used Fletcher’s risk classification by their clinical risk of malignancy: very low, low, intermediate, or high [3].

## Case presentation

**Figure 1:**
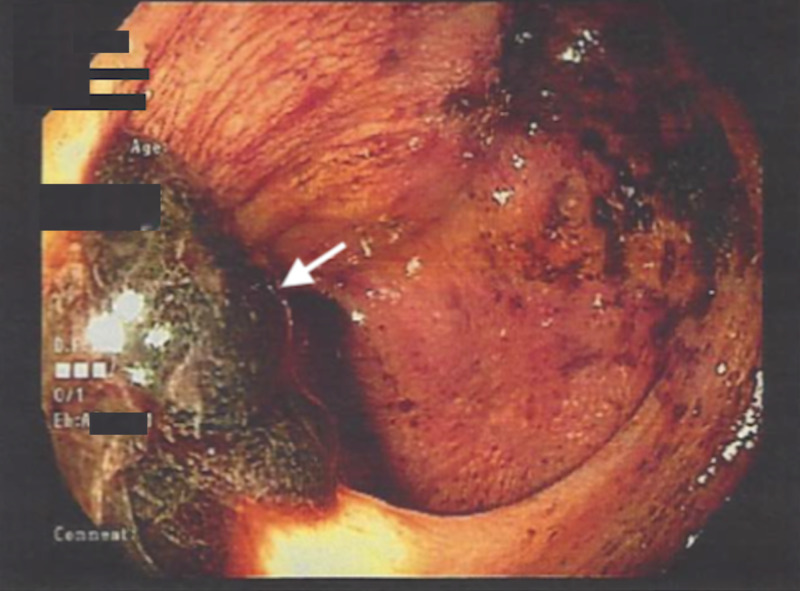
Colonoscopy revealing fresh blood and large blood clots (white arrow) coming from the ileocecal (IC) valve.

**Figure 2:**
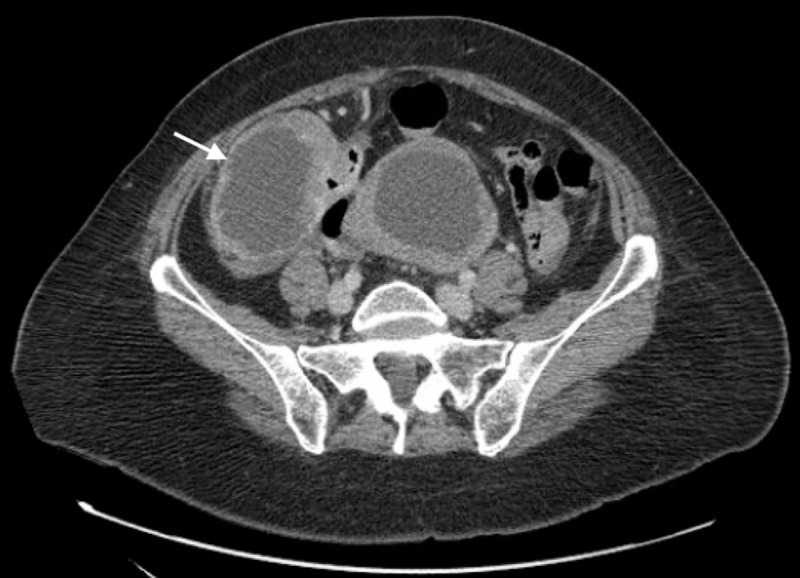
Computed tomography angiogram (CTA) showing a large pelvic, exophytic, necrotic mass with hypervascularity adjacent to the uterine fundus with invasion of the mass into small bowel. Hyperdense material (consistent with blood products) within the lumen of the bowel can be observed.

A 59-year-old woman with a history of gastroesophageal reflux disease (GERD) presented to the emergency room with an one-month history of bright red blood per rectum and lower abdominal pain. She reported one episode of melanic stool. Surgical history was significant for cesarean section, appendectomy, and laparoscopic ovarian cyst excision. Physical examination was unremarkable. Laboratory findings revealed a hemoglobin of 9.4 g/dL and a positive fecal occult blood test. Immunological testing for fecal *Helicobacter Pylori *was positive. The day after admission, an esophagogastroduodenoscopy (EGD) was nondiagnostic and a colonoscopy revealed fresh blood with clots at the terminal ileum (Figure 1), but no evident source of bleeding was identified. As the source of bleeding could not be accurately diagnosed, a computed tomography angiogram (CTA) was obtained. Abdominal CTA revealed a large exophytic, necrotic mass with hypervascularity to the right of the uterine fundus along with extensive vessels within the bowel wall along with multiple necrotic myometrial masses. Air-fluid levels were observed within the colon along with hyperdense material within the right lower quadrant of the bowel wall (Figure 2).

Subsequently, the patient underwent an exploratory laparotomy which demonstrated a mass adherent to the mid-jejunum and terminal ileum along with intimate attachment to the uterosacral ligament and the posterior aspect of the uterus. The mass, distal portion of the distal jejunum, and a portion of the terminal ileum were resected. A total abdominal hysterectomy and salpingo-oophorectomy were also performed. The resected necrotic tumor had a maximal diameter of 21 cm, and no lymph node metastasis was reported. Postoperative pathological examination revealed a high-grade spindle cell variant GIST. The mitotic index was > 5/5 mm^2^ in the high-power field. Immunohistochemical studies revealed CD117 (+), SMA (weak +), CD34 (+), Ki67 (50% strong), PR (weak +), desmin (−), NSE (−), S-100 (−), pankeratin (−), epithelial membrane antigen (−), and estrogen receptor (−). The patient had an uneventful postoperative course and was discharged from the hospital after 10 days. The patient was evaluated by an oncologist who deemed the patient a candidate for adjuvant chemotherapy with imatinib. The patient is doing well at a four-month follow-up with no evidence of recurrent disease.

## Discussion

GISTs account for less than 1% of all gastrointestinal tumors with an incidence rate of 0.68 per 100,000 [4-5]. They are the most common mesenchymal tumors of the gastrointestinal tract [5-6]. GISTs appear to originate from the ICC which are located in the myenteric plexus of the gastrointestinal tract and are responsible for peristalsis [6]. GISTs are largely caused by oncogenic mutations in the tyrosine kinase receptor KIT and/or platelet-derived growth factor receptor-α (PDGFR-α) [7-9].

The anatomical locations of GISTs can vary and are commonly seen in the stomach (55.6%), small bowel (31.8%), colorectal (6%), esophagus (0.7%), and various other locations (5.5%). The median age of presentation is approximately 55-65 years, and they are particularly rare in children [10-11]. Tumor size and mitotic index have been shown to have prognostic value [12-13], and are the current criteria used to stratify risk in the United States National Institutes of Health (NIH) guidelines [14].

Patients with GISTs can often be asymptomatic leading to a missed initial diagnosis. When symptomatic, patients often present with bleeding, anemia, mucosal ulceration, or mass effect. Bleeding is the most common presenting symptom and is attributed to the erosion of the gastrointestinal tract lumen with tumor invasion, resulting in hematemesis, melena, or anemia. However, most patients present with vague symptoms, such as nausea, vomiting, abdominal discomfort, weight loss, or early satiety. At diagnosis, 70% of patients are symptomatic, while 20% are asymptomatic, and 10% are detected at autopsy or incidentally during surgery [15]. Approximately 50% of GISTs are found to be metastatic at the time of diagnosis.

This case offers a unique perspective of GIST’s diagnosis. In cases where a colonoscopy and EGD along with abdominal series are nondiagnostic and when capsule endoscopy/balloon enteroscopy is not available, CT angiography can be considered an alternative imaging procedure for the management of GISTs. In our case, CT angiography provided an exact tumor location preoperatively allowing for appropriate surgical resection.

Surgical intervention is the primary therapeutic option with the goal being complete resection for the nonmetastatic disease. Until recently, GISTs were known for being resistant to chemotherapy. The discovery of activating mutations in these tumors has stimulated the use and development of molecular-targeted therapy. The introduction of tyrosine kinase inhibitors has dramatically changed the management and outcomes of GIST treatment- prolonging recurrence-free survival after surgery and extending overall survival in metastatic or unresectable cases [16]. However, it is difficult to obtain a permanent cure using only tyrosine kinase inhibitors. Therefore, early diagnosis with early surgical resection offers the best clinical outcomes [17-18].

## Conclusions

GISTs are potentially malignant and if left untreated, can be life-threatening. Both tumor size and mitotic index are the most commonly used prognostic criteria that can guide treatment decisions. The recent increase in the diagnosis of GISTs can be attributed to improved imaging techniques. CT angiography is an effective tool for the diagnosis of GISTs, especially in centers where balloon enteroscopy and/or capsule endoscopy are not readily available. Considerable progress has been made with new treatment modalities for the management of these rare tumors. Complete surgical resection of GISTs is the gold standard of primary treatment when possible, with or without the adjunct of molecular-targeted drug therapy.
